# miR-944 inhibits malignant progression of bladder cancer through ATIC/AKT/FOXO3 A axis mediated by SHMT1

**DOI:** 10.1007/s11626-025-01050-1

**Published:** 2025-05-29

**Authors:** Zhiming Liu, Zhao Chen, Haibei Yang, Junning Liu, Maorong Cui, Weisheng Wang

**Affiliations:** Department of Urology, Qujing No.1 Hospital, No.1 Yuanlin Road, Qilin District, Qujing, 655000 Yunnan China

**Keywords:** Bladder cancer, MiR-944, SHMT1, ATIC/AKT/FOXO3 A signaling pathway, Autophagy

## Abstract

To investigate the role of miR-944 in the progression of bladder cancer (BC) and explore its potential as a therapeutic target. In this study, we collected 12 pairs of BC tissues and paracancerous tissues and subcutaneously injected T24 cells into BALB/c nude mice at 1 × 10^6^/mouse to establish the BC animal model for experimental investigation. RT-qPCR and western blot were used to detect the expression of related genes and proteins, and the malignant progression of T24 cells and BC was detected by CCK-8, Transwell, scratch wound, and immunohistochemistry. This study found that miR-944 expression was low in BC clinical samples and cell lines. Overexpression of miR-944 inhibited the proliferation, migration, and invasion of BC cells and inhibited BC tumor growth in vivo. Mechanistically, overexpression of miR-944 downregulated ATIC by inhibiting SHMT1, thereby activating the AKT/FOXO3A signaling pathway and promoting the expression of autophagy-related proteins LC3II/I and Beclin1. At the same time, it can inhibit the expression of epithelial-mesenchymal transition (EMT)-related proteins vimentin, fibronectin, and N-cadherin, ultimately inhibiting the proliferation, migration, and invasion of BC cells, and increasing the apoptosis level of BC cells to improve the development of BC. Our study confirmed that the upregulation of miR-944 may become a new target for the treatment of BC.

## Introduction

Bladder cancer (BC) is the tenth most common cancer in the world and originates from epithelial cells in the bladder (Harsanyi *et al.* 2022). It is one of the most common malignant tumors of the urinary system, with an incidence rate of approximately 3% (Bray *et al.* 2018). The cardinal symptoms of BC include hematuria, frequent urination, urgency, painful urination, and low back pain (Dobruch and Oszczudlowski 2021). Although the cause of BC is still not fully understood, smoking, long-term exposure to certain chemical substances, and chronic cystitis are considered the main risk factors for the pathogenesis of BC (Richters *et al.* 2020). Although significant progress has been made in surgical resection and adjuvant therapy for BC, the prognosis of BC is still poor (Huang *et al.* 2020). Therefore, it is important to explore the molecular mechanisms that regulate the occurrence and development of BC for the treatment of BC.

MicroRNA (miRNA) is a type of single-stranded RNA molecule with a length of about 20–24 nt. It regulates the expression of target genes by directly binding to target gene mRNA and plays key roles in various pathophysiological processes (Vasudevan *et al.* 2007). Studies have shown that miRNAs are involved in the development and progression of BC and can be used as potential biomarkers for prognosis, treatment response, and prescreening of cancerous phenotypes (Dyrskjøt *et al.* 2009, Parizi *et al.* 2020). For example, high expression of miR-612 could inhibit the proliferation, migration, and invasion of BC cells, thus attenuating the development of BC (Li *et al.* 2021). Importantly, in a study of miRNA differential expression between BC and normal bladder tissue, Yin *et al*. (2019) found that miR-944 was highly correlated with the prognosis of BC. In addition, miR-944 is abnormally expressed in cancers of multiple systems, including the nervous, endocrine, respiratory, reproductive, and digestive systems, and miR-944 can regulate the cell cycle, proliferation, migration, invasion, epithelial-mesenchymal transition (EMT) and apoptosis (Shen *et al.* 2022). Therefore, this study investigated the mechanism of action of miR-944 in BC.

Serine hydroxymethyltransferases (SHMTs) are key proteins that regulate carbon (methyl) metabolism, including the cytoplasmic isoenzyme (SHMT1) and the mitochondrial isoenzyme (SHMT2), and play a role in metabolic reprogramming in cancers (Hebbring *et al.* 2012). The SHMT isoenzyme is also a key player in metabolic reprogramming in human tumors and is therefore considered an attractive target for cancer therapy (Giardina *et al.* 2015). Many studies have reported that SHMT1 overexpression can lead to cell growth and the development of malignant tumors (Zhao *et al.* 2015). SHMT1 is involved in the occurrence and progression of many cancers, including gastric cancer (Zhao *et al.* 2020), thymic epithelial tumors (Ku *et al.* 2020), pancreatic cancer (Chittiboyina *et al.* 2018), and hepatocellular carcinoma (Dou *et al.* 2019). These results indicate that SHMT1 has an important regulatory role in the development and progression of cancers, but the mechanism of SHMT1 in BC remains unclear.

In addition, 5-aminoimidazole-4-carboxamide ribonucleotide formyltransferase/IMP cyclohydrolase (ATIC) acts as an enzyme catalyzing the last two reactions in the purine biosynthetic pathway (Verma *et al.* 2017) and has an important regulatory effect on the progression and development of cancers. For example, ATIC can promote the growth, migration, and invasion of lung adenocarcinoma cells (Niu *et al.* 2022). Moreover, ATIC also has an important regulatory role in bladder inflammatory myofibroblast tumors (Debiec-Rychter *et al.* 2003). In addition, ATIC can also promote the progression of HCC through the AKT/FOXO3 signaling pathway (Zhang *et al.* 2021). The AKT/FOXO3 A signaling pathway is also involved in the regulation of BC cell migration and invasion (Su *et al*. 2023). Therefore, we speculated that the ATIC/AKT/FOXO3 A signaling pathway may be involved in the regulation of the process of BC.

In summary, the purpose of this study was to investigate in depth the mechanism of action of miR-944 in the regulation of BC progression through the SHMT1/ATIC/AKT/FOXO3 A axis and to provide a theoretical basis for the development of novel treatments for BC.

## Methods

### Clinical sample collection

Twelve pairs of BC tissues and paracancerous tissues were collected from the hospital and immediately stored in − 80°C liquid nitrogen. None of the patients received chemotherapy or radiotherapy before tumor resection. Informed consent was obtained from all individual participants prior to any study-related procedure. The characteristics of BC patients are shown in Table 1..

**Table 1.** Characteristics of patients with BC

Characteristic	Total = 12	Percentage (%)
Age (years)		
> 72 ≤ 72	6 6	50.0 50.0
Gender		
Male Female	11 1	91.7 8.3
Histological grade		
Low High	5 7	41.7 58.3
TNM stage		
Ι ΙΙ ΙΙΙ IV	3 4 3 2	25.0 33.3 25.0 16.7
Lymph nodes metastasis		
Positive Negative	4 8	33.3 66.7

### Cell culture

The cell lines used in this experiment, normal human bladder epithelial cells (SV-HUC-1 cells) and bladder cancer cells (RT-4 cells, KK47 cells, T24 cells, 5637 cells, HT-1376 cells), were all purchased from Wuhan Pricella Biotechnology Co., Ltd. Wuhan, China. The cells were free of mycoplasma infection, as identified by STR. All cells were cultured in McCoy’s 5 A (modified) medium (Gibco, Invitrogen, Waltham, CA) supplemented with 10% fetal calf serum, 1% penicillin, and streptomycin in an incubator at 37°C and 5% CO_2_. Routine culture and subculture were performed when the cell density reached 90%. To detect the effect of the AKT signaling pathway, we added 10 μM SC79 (AKT activator) to the T24 cells in the groups.

### Animals and experiments

Thirty BALB/c nude mice (weight, 14–16 g) aged 4–6 wk were obtained from the Animal Experiment Center of Kunming Medical University. After the experimental animals were adapted for 1 wk, they were randomly divided into 3 groups (*n* = 10 each). Normal or T24 cells transfected with miR-944 mimic or oe-SHMT1 were subcutaneously injected into BALB/c nude mice at a cell number of 1 × 10^7^ per nude mouse. From day 5, tumor size was measured every 3 d, and the tumor volume was calculated as (length × width^2^)/2. After 20 d, the animals were euthanized, and the tumor tissues were collected for the measurement of relevant indicators.

### Lentiviral transfection

Lentiviral vectors were purchased from Hunan Fenghui Biotechnology Co., Ltd. Changsha, China for lentiviral transfection. Eighteen to twenty-four h before transfection, adherent cells were spread into 24-well plates at 1 × 10^5^/well. Polybrene was added to the level of 6 μg/mL, and an appropriate amount of virus was added to the suspended cells at 2 × 10^5^/mL, mixed evenly, and incubated at 37°C for 4 h. Then, 2 mL of fresh medium was added to dilute the polybrene. Culture was continued for 3–4 d, and then the cells were passaged or the medium was changed, depending on the cell growth status.

### Dual-luciferase experiment

The targeted binding sites of miR-944 and SHMT1 were predicted using the bioinformatics website https://starbase.sysu.edu.cn/. The SHMT1 3′-UTR containing the miR-944 binding site was cloned and inserted into the pGL3 vector (Promega, Madison, WI) to construct the wild-type SHMT1 vector (WT). Mutant SHMT1 vectors (MUT) were generated using a site-directed mutagenesis kit (Stratagene, La Jolla, CA). WT or MUT and overexpression of miR-944 or a negative control were transfected into 293 T cells using Lipofectamine 3000. After 48 h, luciferase activity was detected using a dual-luciferase reporter assay system (Promega).

### Real-time quantitative PCR (RT-qPCR)

Total RNA from normal human bladder epithelial cells, bladder cancer cells, and clinical samples was extracted using TRIzol reagent (Invitrogen, Carlsbad, CA). RNA was reverse-transcribed into cDNA using the First-Strand cDNA synthesis kit (Genenode, Wuhan, China), followed by real-time fluorescence quantification using SYBR Green. Real-time fluorescence quantitative PCR was performed using a PCR kit (Solarbio, Beijing, China) using U6 as an internal reference, and results were calculated using the 2^−ΔΔCt^ method (Livak and Schmittgen 2001). Details of the primer sequences are shown in Table 2..

**Table 2.** RT-qPCR primer sequences

Genes	Primer	Sequence
miR-944	Forward	5′-GCGCGAAATTATTGTACATCG-3′
	Reverse	5′-AGTGCAGGGTCCGAGGTATT-3′
U6	Forward	5′-CTCGCTTCGGCAGCACA-3′
	Reverse	5′-AACGCTTCACGAATTTGCGT-3′

### Western blot

Total proteins from normal human bladder epithelial cells, BC cells, clinical specimens, and cancer tissues of nude mice were extracted, separated by 10% SDS-PAGE, and transferred to polyvinylidene fluoride (PVDF) membranes (Millipore, Burlington, MA). After the membrane was blocked with 5% skim milk for 2 h at room temperature, diluted primary antibody was added and incubated at 4°C overnight. Then, it was conjugated with HRP secondary antibody (1:2000, cat. no. ab205718, Abcam, Cambridge, UK) at room temperature for 1 h. Anti-GAPDH antibody (1:1000, cat. no. ab181602, Abcam) was used as a control. Cell exposure and observation were performed using ECL chemiluminescence solution (Biosciences, Cincinnati, OH). Protein band analysis was performed using ImageJ. The primary antibody information is as follows: anti-SHMT1 (1:1000, cat. no. ab186130, Abcam), anti-E-cadherin (1:10,000, cat. no. ab40772, Abcam), anti-vimentin (1: 5000, cat. no. ab92547, Abcam), anti-fibronectin (1:1000, cat. no. ab2413, Abcam), anti-N-cadherin (1:1000, cat. no. ab76011, Abcam), anti-ATIC (1:2000, A304-272 A, Thermo Fisher Scientific, Waltham, MA), anti-AKT (1:25,000, cat. no. ab81283, Abcam), anti-FOXO3 A (1:1000, cat. no. ab109629, Abcam), anti-Beclin1 (1:2000, cat. no. ab207612, Abcam), anti-p62 (1:1000, cat. no. ab109012, Abcam), anti-LC3B (1:1000, cat. no. ab192890, Abcam).

### Detection of cell proliferation by CCK-8

Detection was performed using a CCK-8 detection kit (Beyotime, Shanghai, China). T24 cells were seeded in 96-well plates at a concentration of 5 × 10^3^ cells/well. They were grouped according to the experimental requirements, and three replicate wells were set up for each group. After performing the experiment according to the requirements of each group, the original culture medium was discarded, and after the addition of new 100 μL cell culture medium, 10 μL CCK-8 solution (Beyotime, Beijing, China) was added and incubated at 37°C in the dark for 2 h. All absorbance values were measured at 450 nm using a microplate reader.

### Scratch experiment

T24 cells in each group in the logarithmic growth phase were collected. After the cells were normally digested and passaged, the cells were seeded on 24-well plates. When the cell density reached 90%, they were scratched with a pipette tip. After the cells were cultured for 24 h, cell migration was observed under a microscope and photographed.

### Transwell experiment

Cell invasion experiments were performed in Transwell wells (Corning, Corning, NY). T24 cells in each group were added to serum-free McCoy’s 5 A medium to adjust the concentration to 1 × 10^5^ cells/mL. Two hundred microliters of cell suspension was added to the upper chamber of a Matrigel-coated Transwell. In the lower chamber of the 24-well plate, 600 μL of McCoy’s 5 A medium containing 10% fetal calf serum was added. After 24 h of culture, the cells in the lower chamber were fixed with 4% paraformaldehyde, stained with crystal violet (Solarbio, Beijing, China), and observed under an inverted microscope (Olympus, Tokyo, Japan) The number of cells at fixed positions in each well was observed, and five fields were selected to count and take pictures.

### Colony formation experiment

T24 cells were seeded in 6-well plates at a density of 1 × 10^3^ cells/well and then cultured continuously for 2 wk to form colonies. Five milliliters of 4% paraformaldehyde was added to each well and incubated for 15 min. Subsequently, colonies were fixed with methanol and stained for 15 min with a Giemsa staining kit (Solarbio, Beijing, China). After washing with tap water, colony counting was performed (Du *et al.* 2018), and the experiment was repeated three times.

### Coimmunoprecipitation

T24 cells were harvested and lysed in IP lysis buffer, and the supernatant was collected by centrifugation. Protein A/G Sepharose (Santa Cruz Biotechnology, Santa Cruz, CA) was incubated with anti-SHMT1 antibody (1:200, cat. no. ab186130, Abcam) and anti-ATIC antibody (1:200, A304-272 A, Thermo Fisher Scientific, Waltham, MA). All IPs were preincubated at 4°C for 60 min with gentle shaking; all IPs were incubated at 4°C overnight with slow shaking; and the beads were collected by centrifugation and washed three times with lysis buffer. The immunoprecipitates were subjected to western blot analysis.

### Monodansylcadaverine (MDC) Labeling

A cell autophagy staining detection kit (MDC method) was purchased from Shanghai Beyotime (cat. no. C3018S). After transfected T24 cells from each group were seeded in 24-well culture plates at 3 × 10^4^ cells per well, 250 µL of MDC (1 ×) was added to the cells for 48 h. Then, the cells were incubated at 37°C and 5% CO_2_ in the dark for 30 min. The MDC staining solution was aspirated off, the cells were washed three times with assay buffer, and then 1 mL of assay buffer was added. The samples were excited with UV excitation light under a fluorescence microscope, and green fluorescence was observed. Alternatively, fluorescence detection was performed using a fluorescence microplate reader at a wavelength of 335 nm.

### Immunohistochemical staining for ki-67

Paraffin-embedded bladder cancer tumor bodies were deparaffinized and rehydrated, and then antigen retrieval was performed in 0.01 M citrate buffer (pH 6.0). Slides were incubated with ki-67 (1:100, cat. no. ab15580, Abcam) at 4°C overnight, and immunodetection was performed using HRP secondary antibody and DAB chromogenic reagent. The results were photographed under a microscope (NiKon, Tokyo, Japan), and quantification of IHC staining density was measured using ImageJ software.

### Detection of apoptosis levels

Flow cytometry was used to determine the level of surface apoptosis, and T24 cells were first harvested, digested with trypsin, and washed with PBS. Cells were stained according to the operating instructions of the Annexin V-FITC/PI Apoptosis Detection kit (Invitrogen, Carlsbad, CA) and incubated at 25°C in the dark for 15 min. After the completion of incubation, the level of apoptosis was measured by flow cytometry (FACScan, Temecula, CA).

### Statistical analysis

All experimental data in this paper are expressed as the mean ± standard deviation (mean ± SD). The data were analyzed and graphed using GraphPad Prism 7. *t* tests were used for comparison between two groups, and one- or two-way analysis of variance (ANOVA) was used for multigroup comparisons, followed by Tukey’s HSD test. *P* < 0.05 indicated that the difference was statistically significant.

## Results

### Differential expression of miR-944 and SHMT1 in BC

**Figure 1.**
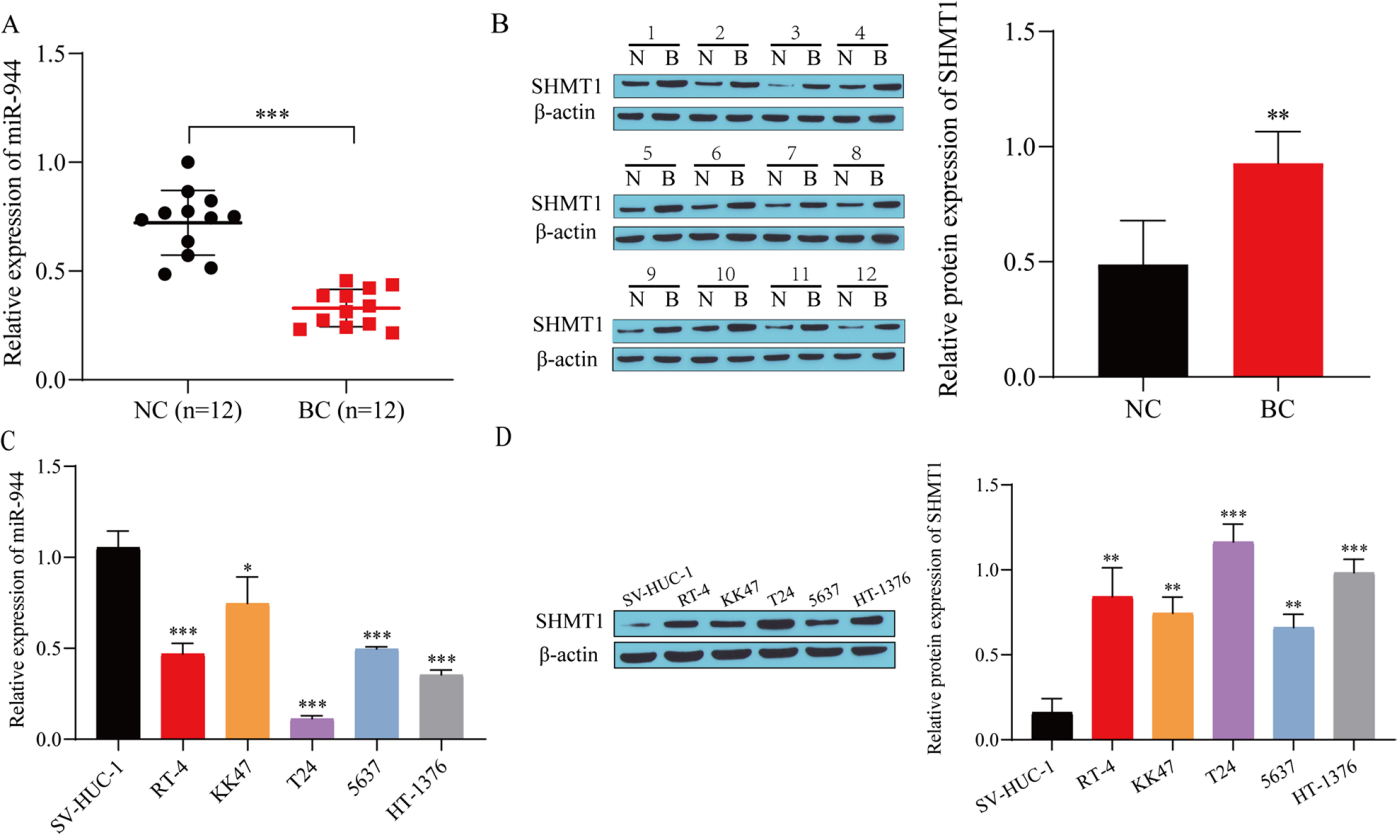
Differential expression of miR-944 and SHMT1 in BC. (*A*) miR-944 expression in clinical samples detected by RT-qPCR. (*B*) SHMT1 expression in clinical samples detected by western blot. (*C*) miR-944 expression in cell lines detected by RT-qPCR. (*D*) SHMT1 expression in cell lines detected by western blot expression. ^*^P < 0.05, ^**^P < 0.01, ^***^P < 0.001 vs. NC or SV-HUC-1.

We first detected the expression of miR-944 and SHMT1 in clinical samples. Compared with paracancerous tissues, miR-944 expression was low in BC tissues (Fig. 1*A*), while SHMT1 expression was high (Fig. 1*B*). Next, the expression of miR-944 and SHMT1 in cells was detected. Compared with SV-HUC-1 cells, miR-944 had low expression in BC cell lines and the lowest expression in T24 cells (Fig. 1*C*), while SHMT1 was highly expressed, with the highest expression in T24 cells (Fig. 1*D*). Therefore, we chose T24 cells for further investigation in subsequent experiments.

### Knockdown of SHMT1 inhibits the malignant progression of BC

**Figure 2.**
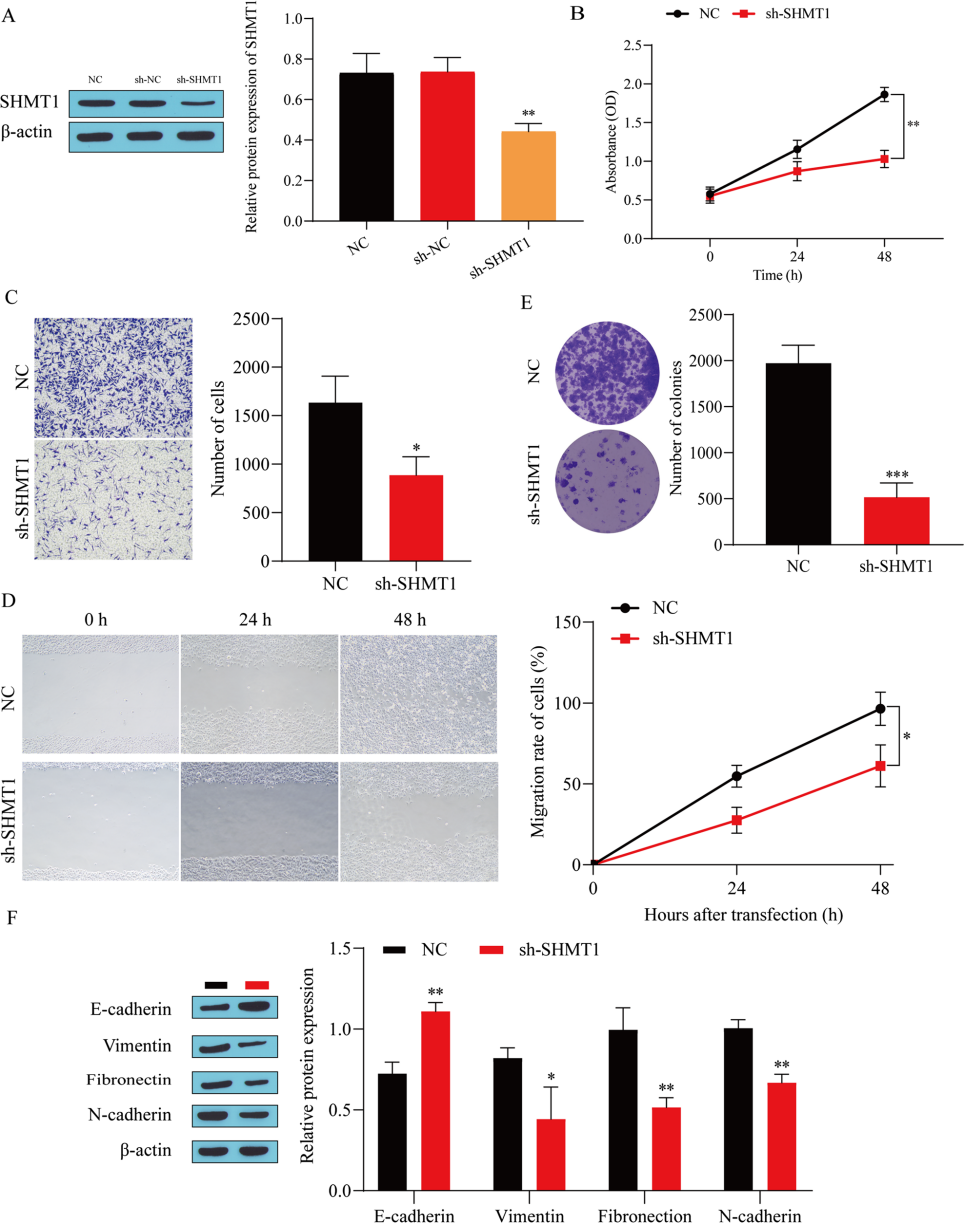
Knockdown of SHMT1 inhibits the malignant progression of BC. (*A*) SHMT1 transfection efficiency detected by western blot. (*B*) Cell viability detected by CCK-8. (*C*) Cell invasion ability detected by Transwell. (*D*) Cell migration ability detected by scratch experiment. (*E*) Colony formation experiment. (*F*) Western blot was used to detect the expression of E-cadherin, vimentin, fibronectin, and N-cadherin. ^*^P < 0.05, ^**^P < 0.01, ^***^P < 0.001 vs. NC.

Next, we used lentiviral infection to transfect sh-SHMT1 into T24 cells to further explore the effect of SHMT1 on the malignant progression of BC. First, the transfection efficiency of SHMT1 was detected. The results showed that the expression level of SHMT1 was significantly downregulated in the sh-SHMT1 group compared with the NC group (Fig. 2*A*), indicating that sh-SHMT1 transfection was successful. Next, the growth status of T24 cells was detected. The results showed that knocking down SHMT1 in T24 cells could significantly inhibit the viability (Fig. 2*B*), invasion (Fig. 2*C*), migration (Fig. 2*D*), and proliferation (Fig. 2*E*) of T24 cells. EMT plays a key role in tumor progression (Matoba *et al.* 2017). Therefore, we detected the expression of EMT-related proteins. The results showed that knocking down SHMT1 significantly promoted the expression of E-cadherin and inhibited the expression of vimentin, fibronectin, and N-cadherin (Fig. 2*F*). These results indicate that knocking down SHMT1 can significantly inhibit the proliferation, invasion, migration, and EMT of BC cells.

### miR-944 targeted the regulation of SHMT1 expression

**Figure 3.**
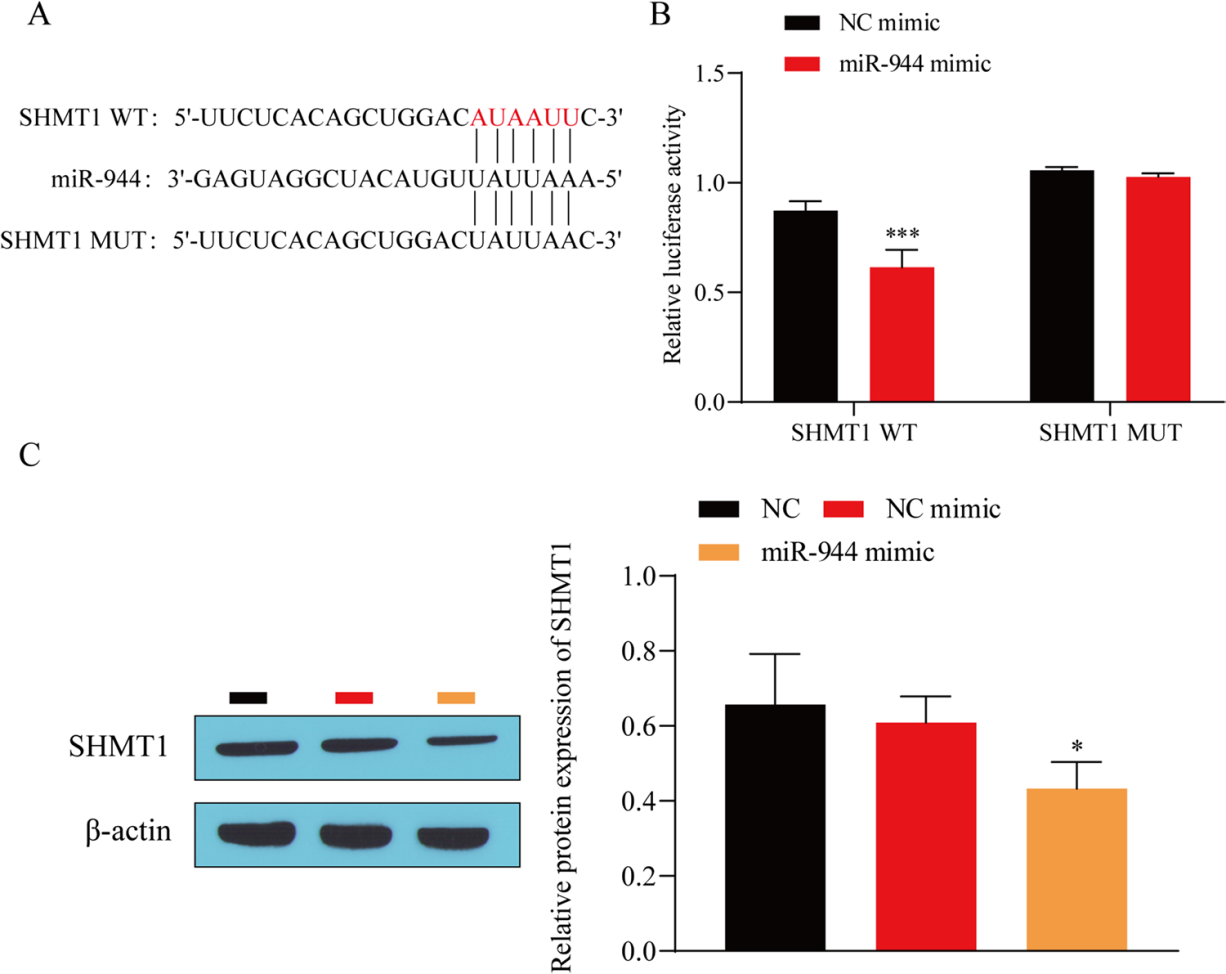
miR-944 targeted the regulation of SHMT1 expression. (*A*) miR-944 and SHMT1 targeting binding sites. (*B*) Dual-luciferase reporter genes validated the targeting relationship between miR-944 and SHMT1. (*C*) Western blot detection of SHMT1 expression. ^*^P < 0.05, ^**^P < 0.01 vs. NC mimic or NC.

Since miR-944 expression is low in BC and SHMT1 is highly expressed, we speculated that there may be mutual regulatory effects between miR-944 and SHMT1. The result was as expected. Analysis of the biological information database StarBase (http://starbase.sysu.edu.cn/) revealed that miR-944 and SHMT1 have mutual binding sites (Fig. 3*A*). Gene reporter experiments and western blot experiments confirmed that miR-944 could target and negatively regulate SHMT1 expression (Fig. 3*B*, *C*). These results indicate that miR-944 can target and negatively regulate SHMT1 expression.

### Overexpression of miR-944 inhibited the malignant progression of BC by regulating SHMT1

**Figure 4.**
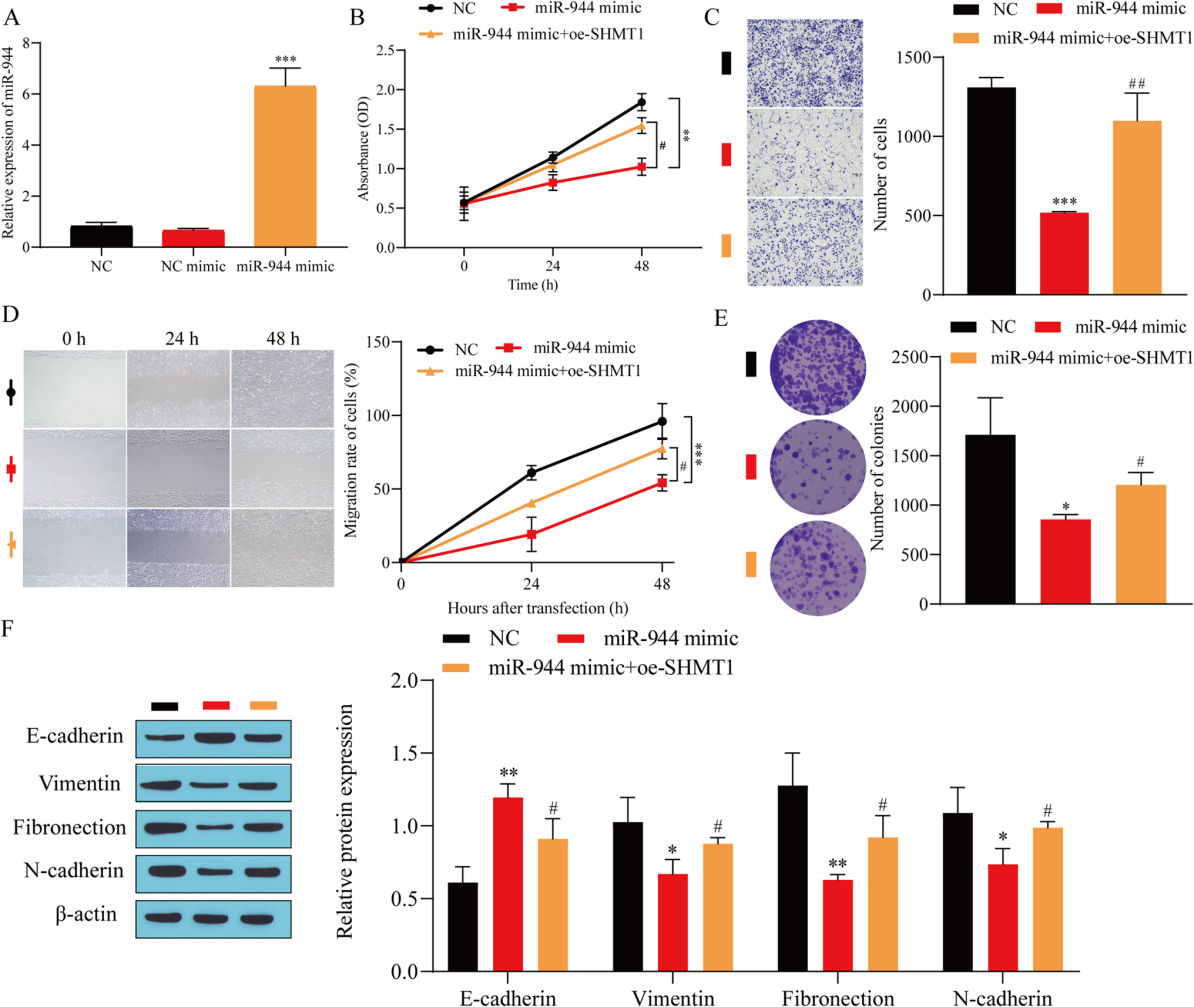
Overexpression of miR-944 inhibits the malignant progression of BC by regulating SHMT1. (*A*) miR-944 transfection efficiency detected by RT-qPCR. (*B*) Cell viability detected by CCK-8. (*C*) Cell invasion ability detected by Transwell. (*D*) Cell migration ability detected by scratch experiment. (*E*) Colony formation experiment. (*F*) Western blot detection of the expression of E-cadherin, vimentin, fibronectin, and N-cadherin. ^*^P < 0.05, ^**^P < 0.01, ^***^P < 0.001 vs. NC; ^#^*P* < 0.05, ^##^*P* < 0.01, ^###^*P* < 0.001 vs. miR-944 mimic.

To explore the effect of miR-944-targeted regulation of SHMT1 on the malignant progression of BC cells, we transfected a miR-944 mimic into T24 cells and detected the transfection efficiency. Compared with the NC group, the miR-944 mimic group had a significantly upregulated miR-944 expression level (Fig. 4*A*), suggesting that the miR-944 mimic was successfully transfected. Next, the growth status of T24 cells was detected. The results showed that overexpression of miR-944 could significantly inhibit the viability, invasion, migration, and proliferation of T24 cells, while further overexpression of SHMT1 increased the cell viability of T24 cells (Fig. 4*B*), invasion (Fig. 4*C*), migration (Fig. 4*D*), and proliferation (Fig. 4*E*). Finally, the expression of EMT-related proteins was detected. The results showed that, compared with the NC group, the overexpression of miR-944 significantly promoted the expression of E-cadherin and inhibited the expression of vimentin, fibronectin, and N-cadherin. Further overexpression of SHMT1 partially reversed the effect of miR-944 overexpression (Fig. 4*F*). These results indicated that miR-944 suppressed the malignant progression of BC cells through the inhibition of SHMT1.

### SHMT1 inhibits the malignant progression of BC by targeting the AKT/FOXO3 A pathway through ATIC

**Figure 5.**
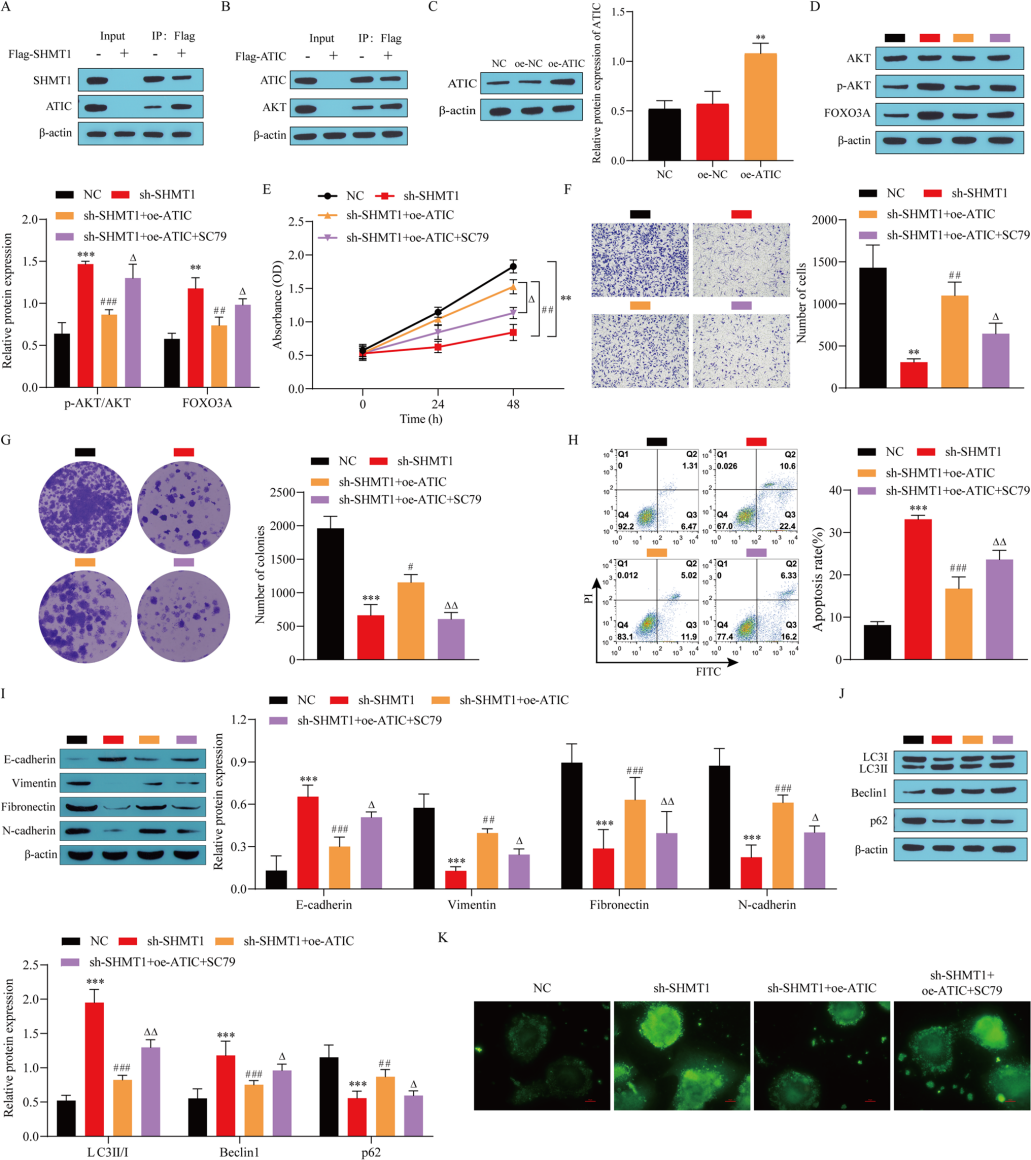
SHMT1 inhibits the malignant progression of BC by targeting the AKT/FOXO3 A pathway through ATIC. (*A*) Coimmunoprecipitation to verify the interaction between SHMT1 and ATIC. (*B*) Coimmunoprecipitation to verify the interaction between ATIC and AKT. (*C*) Western blot detection of ATIC transfection efficiency. (*D*) Western blot detection of AKT, p-AKT, and FOXO3 A expression. (*E*) Cell viability detected by CCK-8. (*F*) Cell invasion ability detected by Transwell. (*G*) Colony formation experiment. (*H*) Cell apoptosis was detected by flow cytometry. (*I*) Western blot was used to detect the expression of E-cadherin, vimentin, fibronectin, and N-cadherin. (*J*) The expression of Beclin1, p62, and LC3 detected by western blot. *K* Autophagy detected by MDC. ^*^P < 0.05, ^**^P < 0.01, ^***^P < 0.001 vs. NC; ^#^*P* < 0.05, ^##^*P* < 0.01, ^###^*P* < 0.001 vs. sh-SHMT1; ^△^*P* < 0.05, ^△Δ^*P* < 0.01 vs. sh-SHMT1 + oe-ATIC.

We further discussed the specific molecular mechanisms by which SHMT1 affects the malignant progression of BC cells. First, the interaction between SHMT1 and ATIC, as well as ATIC and AKT, was verified by coimmunoprecipitation. The results showed that there was an interaction between SHMT1 and ATIC, and there was also an interaction between ATIC and AKT in T24 cells (Fig. 5*A*, *B*). Using lentiviral infection, oe-ATIC was transfected into T24 cells, and the transfection efficiency was determined. Compared with that in the NC group, the expression level of ATIC was significantly upregulated in the oe-ATIC group (Fig. 5*C*), suggesting that oe-ATIC transfection was successful. Next, the expression of AKT/p-AKT/FOXO3 A was detected. Compared with the NC group, SHMT1 knockdown promoted the expression of p-AKT and FOXO3 A proteins. Further overexpression of ATIC repressed the expression of p-AKT and FOXO3 A proteins, which was significantly upregulated after the addition of SC79 (AKT activator) (Fig. 5*D*). In addition, the results of the T24 cell growth status assay showed that the overexpression of ATIC significantly increased the cell viability of T24 cells, promoted their invasion and proliferation, and decreased their apoptosis level compared with the sh-SHMT1 group, while the effect of the overexpression of ATIC was reversed by the addition of SC79 (Fig. 5*E*, *H*). The expression of EMT-related proteins was also detected, and the results also showed that ATIC overexpression significantly inhibited the expression of E-cadherin and promoted the expression of vimentin, fibronectin, and N-cadherin compared with the sh-SHMT1 group. However, the addition of SC79 reversed the effect of ATIC overexpression (Fig. 5*I*). Western blot showed that the knockdown of SHMT1 promoted the expression of LC3II/I and Beclin1 and inhibited the expression of p62 compared with the NC group. Further overexpression of ATIC partially reversed the effect of the knockdown of SHMT1, which was restored after the addition of SC79 (Fig. 5*J*). Detection of autophagy in MDCs showed that knocking down SHMT1 promoted autophagy, further overexpression of ATIC inhibited autophagy, and the addition of SC79 promoted autophagy (Fig. 5*K*). These results suggest that SHMT1 inhibits the AKT/FOXO3 A signaling pathway by promoting ATIC to promote the proliferation, invasion, and EMT of BC cells and reduce their apoptosis level, thereby promoting the malignant progression of bladder cancer. In addition, SHMT1 may also participate in the malignant progression of BC cells by affecting the expression of autophagy-related proteins and the level of autophagy.

### miR-944 targeted the regulation of SHMT1 to inhibit the malignant progression of BC through the ATIC/AKT/FOXO3 A axis

**Figure 6.**
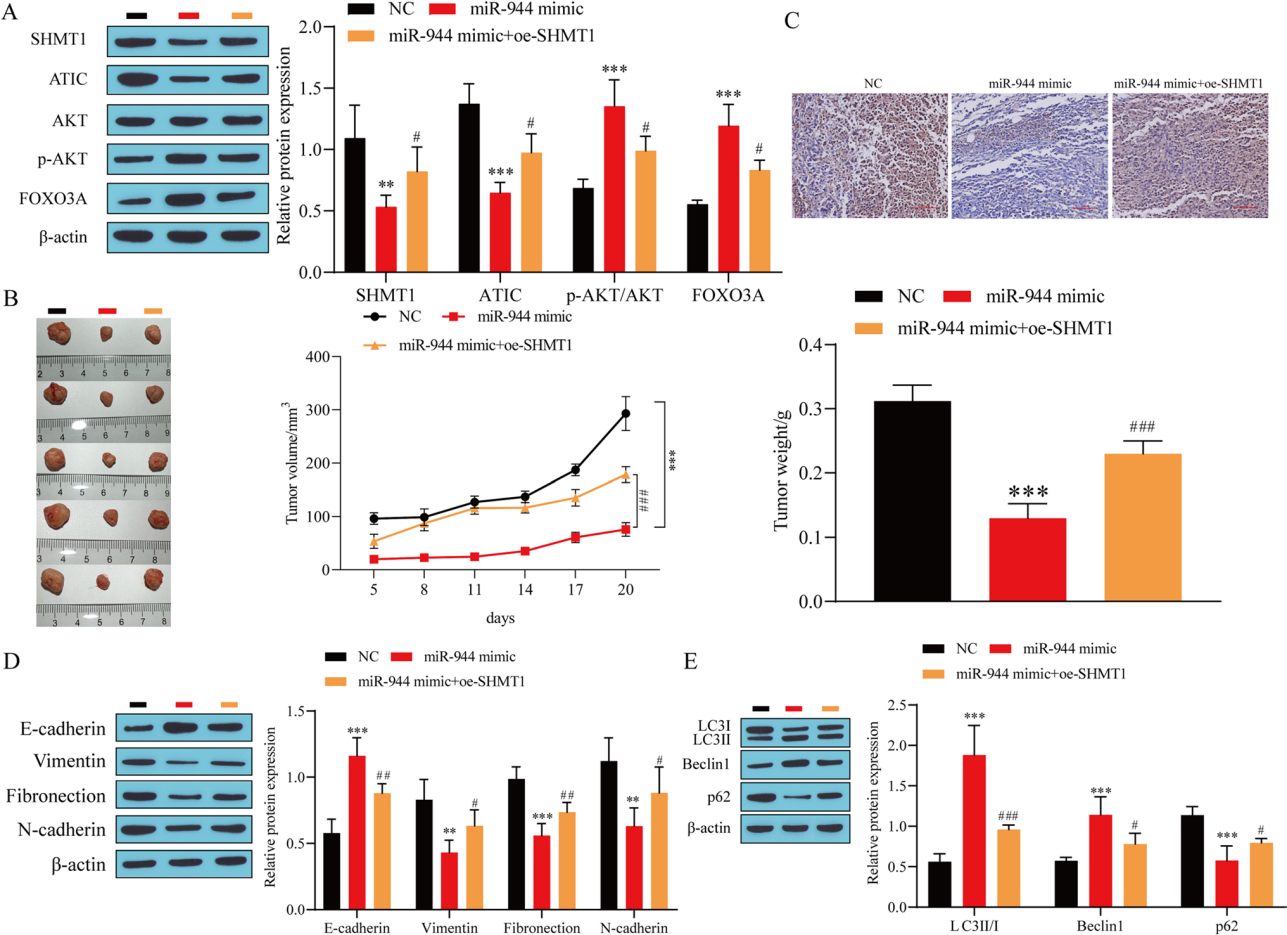
miR-944 targeted the regulation of SHMT1 to inhibit the malignant progression of BC through the ATIC/AKT/FOXO3 A axis. (*A*) The expression of SHMT1, ATIC, P-AKT, and FOXO3 A detected by western blot. (*B*) The volume and quality monitoring of the tumor mass of BC in nude mice. **(***C*) The expression of ki-67 detected by immunohistochemical staining. (*D*) Western blot was used to detect the expression of E-cadherin, vimentin, fibronectin, and N-cadherin. (*E*) The expression of Beclin1, p62, and LC3 detected by western blot. ^**^P < 0.01, ^***^P < 0.001 vs. NC; ^#^*P* < 0.05, ^##^*P* < 0.01, ^###^*P* < 0.001 vs. miR-944 mimic.

To validate the above cell experiments, we constructed a BC nude mouse model to further explore whether miR-944 affects the progression of BC through the SHMT1/ATIC/AKT/FOXO3 A axis. First, the results of western blot analysis showed that compared with the NC group, miR-944 overexpression significantly inhibited the expression of SHMT1 and ATIC and promoted the expression of p-AKT and FOXO3 A; overexpression of SHMT1 partially reversed the effect of miR-944 overexpression (Fig. 6*A*). Overexpression of miR-944 significantly inhibited the growth of BC tumors, and further overexpression of SHMT1 promoted the growth of BC tumors (Fig. 6*B*). Next, the results of immunohistochemistry showed that overexpression of miR-944 significantly inhibited the expression of ki-67, and further overexpression of SHMT1 promoted ki-67 expression (Fig. 6*C*). In the end, western blot results showed that overexpression of miR-944 significantly promoted the expression of E-cadherin, LC3II/I, and Beclin1, and inhibited the expression of Vimentin, Fibronectin, N-cadherin, and p62. However, further overexpression of SHMT1 partially reversed the effect of miR-944 overexpression (Fig. 6*D*, *E*). These results indicated that miR-944 suppressed the malignant progression of BC in nude mice by regulating the SHMT1/ATIC/AKT/FOXO3 A axis.

## Discussion

BC is one of the most common malignant tumors in urological tumors. The clinical treatment of BC has a variety of options, including neoadjuvant or adjuvant chemotherapy, radiotherapy, and immunotherapy (Witjes *et al.* 2021). However, these treatment methods all have problems, such as poor overall efficacy and poor prognosis. Therefore, it is still important to look for novel prognostic biomarkers and treatment methods for BC.

It is well known that miRNAs can be used as biomarkers for cancer diagnosis and treatment response prediction (Bertoli *et al.* 2015; Sawaki *et al.* 2018). In recent years, many miRNAs have been found to be involved in the regulation of the progression of BC and to affect the treatment efficiency of BC. For example, Li *et al.* (2023) found that upregulation of miR-3960 could inhibit the progression of BC cells by inhibiting the proliferation and promoting the apoptosis of BC cells. Fu *et al.* (2019) showed that overexpression of miR-3622a could significantly increase the proliferation and invasion ability of BC cells, thereby accelerating the development of BC. Existing studies have shown that miR-944 is significantly associated with the prognosis of BC (Yin *et al.* 2019). However, its specific mechanism of action in BC remains unclear. Therefore, our study mainly focused on miR-944. We found that miR-944 was significantly downregulated in BC clinical samples and cell lines. Overexpression of miR-944 not only inhibited the proliferation, migration, and invasion of BC cells but also inhibited tumor growth in nude mice. We built on the study by Yin *et al*. (2019) and found miR-944 as a novel molecule for alleviating BC progression.

One of the signs of cancer is metabolic disorders, and more and more studies have taken it as a potential therapeutic target for cancer. SHMT1, one of the key enzymes in the synthetic pathway of serine/glycine and monocarbon metabolism, has been proven to be a new driver gene in human cancer (Koo and Yoon 2015). As Gupta *et al.* (2017) found in ovarian cancer, SHMT1 can promote the expression of pro-cancer factors through sialic acid, thereby promoting the ability of ovarian cancer tumor growth and migration. Paone *et al.* (2014) found in lung cancer that knocking down SHMT1 could inhibit the progression of lung cancer by inducing cell cycle arrest and p53-dependent apoptosis. Importantly, SHMT1 is also associated with the risk of BC (Moore *et al.* 2007). Therefore, we explored the function of SHMT1 in BC and found that SHMT1 was significantly highly expressed in BC clinical samples and cell lines and that knocking down SHMT1 could inhibit the malignant biological behavior and EMT of BC cells. Our study and previous studies both showed that SHMT1 can promote the progression of cancer. In addition, the function of SHMT1 in cancer is also regulated by miRNA (Yang *et al.* 2019). Therefore, we verified the interaction between miR-944 and SHMT1 and found that miR-944 could target and negatively regulate the expression of SHMT1, and the inhibitory effect of miR-944 overexpression on BC progression could be reversed by SHMT1 overexpression. Our study showed for the first time that miR-944 can suppress BC progression through the inhibition of SHMT1.

In exploring the downstream regulatory mechanisms of SHMT1, some studies have found that ATIC is associated with the prognosis and immunotherapy sensitivity of BC (Liu *et al.* 2023). Moreover, the AKT/FOXO3 A signaling pathway is also involved in the regulation of the progression of BC (Zhuo *et al.* 2019). Importantly, miRNA can affect the process of BC by regulating AKT/FOXO3 A signaling pathway. For example, Liang *et al*. (2017) found that miR-608 can inhibit the proliferation and cell cycle progression of BC cells by downregulating AKT/FOXO3 A signaling pathway. Chang *et al*. (2020) showed that miR-516a can promote BC migration and invasion by activating the AKT/FOXO3 A signaling pathway. Therefore, we investigated the interaction among SHMT1, ATIC and AKT and found that SHMT1 can bind to ATIC and that ATIC can bind to AKT. These results indicate that SHMT1 regulates the ATIC/AKT/FOXO3 A pathway. We then knocked down SHMT1, overexpressed ATIC and added SC79, an AKT activator, at the cellular level. The results showed that SHMT1 promoted the proliferation, invasion and EMT of BC cells by promoting ATIC to inhibit AKT/FOXO3 A signaling pathway, which was consistent with the above literature. Notably, the apoptosis level of T24 cells was significantly increased in the sh-SHMT1 group, and ATIC overexpression significantly reduced the apoptosis level, while the addition of SC79 reversed the effect of ATIC overexpression. Considering that the proliferation and invasion ability of BC cells will be affected after apoptosis, combined with the inhibition of AKT/FOXO3 A signaling pathway and the changes of related biological behaviors, it is suggested that the proliferation and invasion of BC cells may be the result of both direct targeting AKT/FOXO3 A pathway by SHMT1 and indirect inducing apoptosis. The effect of SHMT1 knockdown–induced apoptosis on cell proliferation and invasion needs to be further explored. Although our study has some limitations, the available data still support that SHMT1 inhibits tumor cell proliferation, invasion and EMT and increases the level of apoptosis by inhibiting AKT/FOXO3 A signaling pathway.

In addition, the AKT/FOXO3 A signaling pathway can regulate cancer autophagy in vitro and in *vivo*, thereby affecting cancer proliferation and apoptosis (Abdullah *et al.* 2021). Autophagy is a process of self-degradation and recycling of cells, which maintains the stability of the intracellular environment by forming autophagosomes to remove damaged organelles and misfolded proteins within cells (Kamal *et al.* 2021). Recent studies have shown that autophagy plays a complex role in tumor occurrence and development, which can not only promote the growth and survival of tumor cells, but also inhibit the proliferation and invasion of tumor cells (Santana-Codina *et al.* 2017). Autophagy has been shown to have anti-tumor functions. Firstly, autophagy can promote the apoptosis of tumor cells by degrading tumor suppressor proteins and activating pro-apoptotic signaling pathways (Kroemer *et al.* 2010). Secondly, autophagy can remove damaged mitochondria and protein aggregates in cells, reduce oxidative stress and inflammatory response, and thus inhibit the proliferation and invasion of tumor cells (Kotb *et al.* 2016). In addition, autophagy can also eliminate cell damage caused by chemotherapy drugs, thereby enhancing the sensitivity of tumor cells to chemotherapy drugs (Kroemer *et al.* 2010). In BC, Wang *et al.* (2020) elucidated that activation of autophagy and inhibition of EMT contribute to the treatment of BC, and the mechanism of action of autophagy is expected to be used to improve the treatment of BC (Sun *et al.* 2021).In this study, we found that knocking down SHMT1 could activate the AKT/FOXO3 A signaling pathway and promote BC cell autophagy through the inhibition of ATIC, thereby inhibiting the malignant progression of BC cells in vitro and the growth of BC tumors in nude mice. Our study and Liang *et al.* (2017) and Chang *et al.*’s (2020) studies were inconsistent, which might be due to differences in the type, classification, stage, cell environment, and upstream regulatory factors of BC.

In summary, we found that miR-944 downregulated ATIC through the inhibition of SHMT1, thereby activating the AKT/FOXO3 A signaling pathway, promoting autophagy, and inhibiting the proliferation, migration, and invasion of BC cells. However, our study has certain limitations. Firstly, our functional experiments were mainly performed on the T24 cell line. Although the T24 cell line was selected based on its typical low expression of miR-944 and high expression of SHMT1, future studies need to validate these findings in more bladder cancer cell lines such as HT-1376 and 5637 to further improve the reliability and generalization of the findings. Secondly, in order to better understand the role of miR-944 in inhibiting bladder cancer progression, it is necessary to further study the upstream mechanism regulating miR-944 expression and explore the specific mechanism of autophagy in bladder cancer progression in the future. Finally, although we revealed the function of miR-944 through cell and animal experiments, there is still a lack of support from clinical trials. Further studies are needed to apply miR-944 to the treatment of bladder cancer in the future. In conclusion, our study demonstrated that the upregulation of miR-944 was able to inhibit bladder cancer initiation and progression.

## Data Availability

The datasets used and/or analyzed during the current study are available from the corresponding author upon reasonable request.
